# Structure Elucidation and Anti-Tumor Activities of Trichothecenes from Endophytic Fungus *Fusarium* *sporotrichioides*

**DOI:** 10.3390/biom12060778

**Published:** 2022-06-02

**Authors:** Ya-Jing Wang, Nan Ma, Yong-Fu Lu, Si-Yang Dai, Xue Song, Chang Li, Yi Sun, Yue-Hu Pei

**Affiliations:** 1 Department of Medicinal Chemistry and Natural Medicine Chemistry, College of Pharmacy, Harbin Medical University, Harbin 150081, China; w_wangyajing@163.com (Y.-J.W.); 18845646718@163.com (N.M.); yong15765985768@163.com (Y.-F.L.); daisiyangjy@163.com (S.-Y.D.); sunshine13140203@126.com (X.S.); 2 Institute of Chinese Materia Medica, China Academy of Chinese Medical Sciences, Beijing 100700, China

**Keywords:** trichothecene, apoptosis, cell cycle arrest, mitochondrial pathway, anti-tumor

## Abstract

The secondary metabolites of *Fusarium sporotrichioides*, an endophytic fungus with anti-tumor activity isolated from *Rauvolfia yunnanensis* Tsiang, were investigated. Five trichothecenes, including one previously undescribed metabolite, were isolated and identified. Their structures were elucidated by means of extensive spectroscopic methods; the absolute configuration of compound **1** was determined by the ECD method. Surprisingly, 8-n-butyrylneosolaniol (**3**) exhibited stronger anti-tumor activity than T-2 toxin against Huh-7 cell line, with an IC_50_ value of 265.9 nM. 8-n-butyrylneosolaniol (**3**) promoted apoptosis induction in Huh-7 cells. Moreover, cell cycle analysis showed that cell cycle arrest caused by 8-n-butyrylneosolaniol (**3**) at the G2/M phase resulted in cell proliferation inhibition and pro-apoptotic activity. Further studies showed a significant decrease in mitochondrial membrane permeabilization and a significant increase in ROS generation, which led to the activation of caspase cascades and subsequent cleavage of PARP fragments. In conclusion, 8-n-butyrylneosolaniol (**3**) induced cell apoptosis in Huh-7 cells via the mitochondria-mediated apoptotic signaling pathway, which could be a leading compound for anti-tumor agents.

## 1. Introduction

Endophytic microorganisms have been found in virtually every medicinal plant and contribute to their host plant by producing a plethora of substances that provide protection to the plant [1,2]. Endophytic fungi have been viewed as an outstanding source of novel and bioactive natural products because they have produced a number of famous and important compounds [3,4,5]. As our going research on anti-tumor secondary metabolites from endophytic fungi of medicinal plants, one strain *Fusarium sporotrichioides* isolated from *Rauvolfia yunnanensis* Tsiang has attracted our attention because its mycelial extracts showed strong cytotoxicity against A549 cell line (IC_50_ = 15.4 µg/mL). Then, the crude extract of *F.* *sporotrichioides* in solid rice culture was chemically investigated, which led to the isolation of five trichothecenes (**1**–**5**), including one previously undescribed trichothecene named 8-(2-methylbutyryl)-neoso-laniol (**1**).

It is well known that trichothecenes are a large family of mycotoxins produced by several fungal genera, including *Fusarium* [6,7]. Currently, approximately 200 members of the trichothecene family have been identified [8]. Trichothecenes are also a source of contamination in food, which could induce a series of acute and chronic symptoms, such as hypotension, anemia and so on [9]. Nevertheless, some trichothecenes have also been reported to exhibit antifungal, antimalarial, antiviral and phytotoxic activities [9,10,11]. The biological investigation of the five compounds suggested that all of them exhibited cytotoxicities against Huh-7 cells and MRMT-1 cells. Among them, 8-n-butyrylneosolaniol (**3**) exhibited stronger cytotoxic activity against the Huh-7 cell line than the T-2 toxin, with an IC_50_ value of 265.9 nM. Further studies demonstrated **3** could induce cell cycle arrest during the G2/M phase and induce cell apoptosis in Huh-7 cells via the mitochondria-mediated apoptotic signaling pathway. Herein, we report on the isolation, structural determination and biological properties of these compounds. In this work, we aimed to discover potential anti-tumor candidates from endophytic fungi of medicinal plants by using chemical profiles and anti-tumor activity evaluation.

## 2. Materials and Methods

### 2.1. Instruments, Chemicals, and Biochemicals

Optical rotation was measured on NICOLET iS5 (Thermo, Waltham, MA, USA) spectrometer, UV spectrum was recorded on Evolution 220 (Thermo, Waltham, MA, USA) UV/Vis spectrometer. IR spectrum was measured on IR Tracer-100 (Shimadzu, Kyoto, Japan); 1D and 2D-NMR spectra were measured on a Bruker AV-600 spectrometer (CD_3_OD as solvent) (Bruker, Karlsruhe, Germany). HRESIMS data were determined by Waters Vion IMS QTOf mass spectrometer (Waters, Milford, CT, USA). ECD spectra were measured on Bio-logic MOS-450 circular dichrometer (Biologic, Grenoble, France). Open column chromatography (CC) was performed using silica gel (200–300 mesh, Qingdao Haiyang Chemical Goup Corp. Qingdao, China) and ODS (50 µm, YMC, Japan). The samples were purified by semipreparative high-performance liquid chromatography (HPLC) equipped with an LC-20AR system, a UV detector, and an HPLC column (5 µm, 10 × 250 mm, COSMOSIL AR II-C18 column). Dulbecco’s Modified Eagle’s Medium (DMEM) and phosphate-buffered saline (PBS) were purchased from Gibco (Gibco, Carlsbad, CA, USA). T-2 toxin was purchased from Sichuan Weikeqi Biological Technology Co., Ltd. (Chengdu, China). Cytochrome C, caspase 9, cleaved caspase 3, PARP and GAPDH antibodies were supplied from Cell Signaling Technology (CST, Beverly, CA, USA). Cell Counting Kit-8 kit was purchased from Dojindo Laboratories (Kumamoto, Japan). Reactive Oxygen Species Assay Kit was purchased from Shanghai Beyotime Biotechnology Co., Ltd. (Shanghai, China). A mitochondrial membrane potential assay kit with JC-10 was purchased from Solarbio Life Sciences Co., Ltd. (Beijing, China). PI and PI-Annexin V assay kits were purchased from Thermo Fisher (Carlsbad, CA, USA). 

### 2.2. Strain Isolation and Cultivation

The fungal strain was isolated from the inner tissue of the surface-sterilized fruit of *RauwoIfia yunnanensis* Tsiang, which was collected in Yunnan Province in China in 2017. It was identified as *Fusarium sporotrichioides* by its rRNA gene sequence and given the Genbank accession number MT649476.1. A voucher specien (No.luo4-fruit-2i-b) was deposited at the Institute of Chinese Materia Medica, China Academy of Chinese Medical Sciences, Beijing. The strain was fermented on autoclaved rice solid-substrate medium (eighty 500 mL Erlenmeyer flasks, each containing 40 g rice and 60 mL water) for 12 days at 28 °C.

### 2.3. Extraction and Isolation

Following incubation, the solid rice media were extracted three times with EtOAc to give a crude extract (22.3 g), which was suspended in 95% MeOH followed by partition with petroleum ether to afford a 95% MeOH total extract (10.2 g). The extract was fractionated by ODS flash column chromatography (5 × 30 cm) eluting with 2 L of each of 20%, 40%, 60%, 80% and 100% (*v*/*v*) MeOH in H_2_O. The fraction eluted with 60% MeOH was divided into four subfractions (G1–G4) by using CC (silica gel 200–300 mesh, ethyl acetate/methanol (30:1 to 3:1), and then, subfraction G1 was subjected to silica-gel CC, eluted with a dichloromethane/acetone gradient, to yield 14 subfractions (A1–A14). Subfraction A5 was purified by semipreparative HPLC (Cosmosil AR II-C18 column, 250 mm × 10 mm i.d., 5 µm, 3 mL/min) with a gradient elution from 35% to 45% acetonitrile in H_2_O with 0.1% AcOH to afford compounds **2** (t_R_ = 17.6 min, 3.4 mg), **4** (t_R_ = 28.2 min, 5.6 mg), **3** (t_R_ = 29.5 min, 6.8 mg), **1** (t_R_ = 45.5 min, 4.2 mg) and **5** (t_R_ = 49.8 min, 1.8 mg). 

*8-(2-methylbutyryl)-neosolaniol* (**1**). ${\left[\alpha \right]}_{D}^{20}$+59 (c 0.1 MeOH); UV (MeOH) λ_max_ (log) 200 (3.62) nm, 224 (2.89) nm; IR (KBr) 3432, 2959, 1733 and 1256 cm^−1^; CD (MeOH) 220 (Δε −9.62) nm; HRESIMS *m*/*z* [M + Na]^+^ 489.2089 (calcd for C_24_H_34_O_9_Na, 489.2095); For ^1^H NMR and ^13^C NMR spectroscopic data, see Table 1.

### 2.4. Cell Lines and Cell Culture

The human hepatoma Huh-7 cells and rat mammary tumor MRMT-1 cells were purchased from ATCC (Manassas, VA, USA). DMEM medium was purchased from ATCC (Manassas, VA, USA). Penicillin–streptomycin, 0.25% trypsin and fetal bovine serum (FBS) were purchased from Gibco (Grand Island, NY, USA). Cells were cultured in DMEM supplemented with 10% FBS and 1% penicillin–streptomycin in a humidified atmosphere at 37 °C with 5% CO_2_.

### 2.5. Cell Viability Assay

Cell Counting Kit-8 assay (Dojindo Laboratories, Kumamoto, Japan) was conducted to measure the viability of cells. Briefly, a suspension of Huh-7 or MRMT-1 (3 × 10^4^ cells/well) in 100 µL of DMEM with 10% FBS was seeded to 96-well plates and supplemented with different concentrations (4, 20, 100, 500, 1250, 2500 and 5000 nM) of compounds (**1**–**5**). Following treatment of 48 h, 10 µL CCK-8 solution was added to each well and incubated for 4 h at 37 °C. In this experiment, T-2 toxin is a positive control, and blank culture is a negative control. Absorbance was measured at a wavelength of 450 nm using a microplate reader (Perkin-Elmer, MA, USA). Each experiment was performed in triplicate. IC_50_ values were calculated using GraphPad Prism 9.0 (GraphPad Software, Inc., San Diego, CA, USA).

### 2.6. Cell Cycle Assay

The cell cycle was measured by PI staining and flow cytometry analysis. Approximately 1 × 10^5^ cells/mL of Huh-7 cells were seeded into 24-well plates and incubated for 24 h. The cells were treated with 40, 400 and 800 nM of compound **3** for 24 h, harvested and washed once with PBS. Then the cells were incubated with 70% cold ethanol at -20 °C overnight. After fixation, cells were washed twice with cold PBS and centrifuged (12,000 rpm) to discard the supernatant. The pellet was resuspended with PBS and stained with PBS containing 50 µg/mL PI and 100 µg/mL RNase A for 30 min in the dark. The cell cycle phase distribution was analyzed using flow cytometry (Beckman coulter, CA, USA). 

### 2.7. Cell Apoptosis Analysis

The apoptosis induction was detected by the annexin-V/PI staining method. Huh-7 cells (2 × 10^5^ /well) were cultured in 6-well plates for 24 h, then treated with **3** of various concentrations (0, 40, 400 and 800 nM) for 36 h. The test procedure followed the manufacturer’s instructions. Then cells were harvested, washed, and suspended in 500 µL annexin V binding buffer; after that, annexin V-FITC (5 µL) and PI (5 µL) were added. After being stained in the dark for 15 min at room temperature, the cells were analyzed by use of flow cytometry (Beckman coulter, CA, USA).

### 2.8. Mitochondrial Membrane Potential Analysis

The mitochondrial membrane potential (MMP) was determined by JC-10 dye staining. Huh-7 cells were incubated with different concentrations of compound **3** (0, 40, 400 and 800 nM) for 32 h. After removing the culture, the cells were collected by trypsinization, centrifuged (12,000 rpm), and then suspended in a DMEM medium at the concentration of 1 × 10^5^ cells/mL. The cells were kept in the incubator (5% CO_2_) for 20 min after being mixed with JC-10 stain liquid. Finally, the cells were imaged by means of a fluorescence microscope after washing.

### 2.9. Measurement of ROS Generation

The production of ROS was detected by flow cytometry. Briefly, Huh-7 cells were plated in six-well plates and incubated for 24 h. Then cells were treated with various concentrations of **3** (0, 40, 400 and 800 nM) for 32 h. After exposure, DCFH-DA (10 mmol/L) was added and incubated at 37 °C for 30 min in the dark. Then, cells were washed with **3** at doses of 40, 400 and 800 nM for 32 h. Cells were analyzed by flow cytometer (Beckmancoulter, CA, USA). 

### 2.10. Western Blot Analysis

Huh-7 cells were incubated with **3** for whole-cell lysates or lysis buffer NP-40 for cytoplasmic lysates. Then, cells were harvested in lysis buffer RIPA for whole-cell lysates and then incubated for 15 min at 4 °C. After centrifugation (12,000 rpm), the supernatant was obtained for analysis. The protein concentration of the supernatant was determined by BCA (Thermo Fisher, Carlsbad, CA, USA) assay. Supernatants were then mixed with SDS-PAGE sample buffer and boiled for 5 min. Samples were subsequently loaded into each lane of a 10% SDS-PAGE and transferred onto PVDF membranes (Bio-Rad, Philadelphia, PA, USA). Membranes were blocked for 1 h in 5% (*w*/*v*) non-fat milk and then incubated with a primary antibody at room temperature for 1 h. The following antibodies were used: cytochrome C, caspase-9, cleaved caspase-3 and PARP. Having been washed 3 times in TBST, the membranes were incubated with horseradish peroxidase (HRP)-conjugated rabbit IgG (diluted 1:10,000) for 1 h at room temperature and then washed 3 times. Finally, proteins were detected using SuperSignal West Dura ECL substrates (Thermo Fisher, Carlsbad, CA, USA). The load protein was normalized to GAPDH. All experiments were performed in triplicate.

### 2.11. Statistical Analysis

Data are expressed as mean ± standard deviation (SD). Unpaired *t*-tests were performed for comparison between the two groups. One-way analysis of variance (ANOVA) followed by Bonferroni’s post hoc test was used to compare more than two groups. Differences were considered significant at *p* < 0.05.

## 3. Results

### 3.1. Structure Elucidation of Compound ***1***

**Figure 1:**
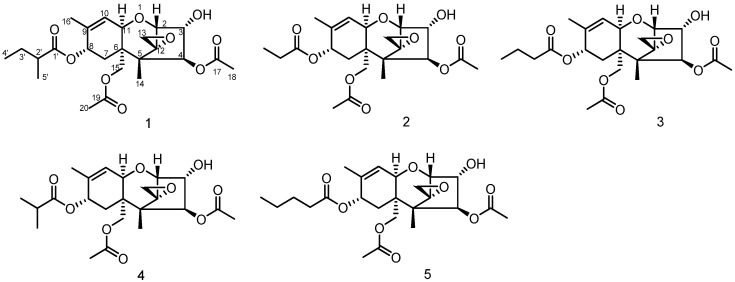
Chemical structures of the identified compounds **1**–**5**.

**Figure 2:**
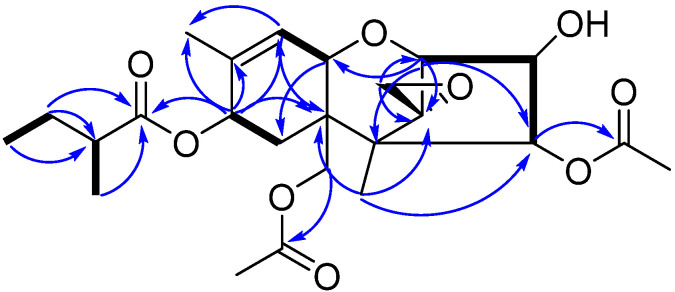
Key ^1^H-^1^H COSY (black solid line) and HMBC (blue single arrow) correlations of **1**.

Compound **1** (Figure 1) was obtained as a white powder with a molecular formula of C_24_H_34_O_9_ based on its HRESIMS data at *m*/*z* 489.2089 [M + Na]^+^ (calcd for C_24_H_34_O_9_Na, 489.2095), indicating eight degrees of unsaturation. The IR spectrum showed absorption bands at 3432 and 1733 cm^−1^, assignable to hydroxyl and ester carbonyl groups. The ^1^H-NMR spectrum of **1** recorded in CD_3_OD showed six methyl signals at δ_H_ 0.72 (s, H-14), 0.92 (t, J = 7.4 Hz, H-4’), 1.14 (d, J = 7.0 Hz, H-5’), 1.74 (s, H-16), 2.05 (s, H-20) and 2.08 (s, H-18); In addition, there was one olefinic proton signal at δ_H_ 5.80 (d, J = 5.8 Hz, H-10) and five oxygenated methine signals at δ_H_ 3.54 (d, J = 4.9 Hz, H-2), 4.22 (dd, J = 4.9, 3.1 Hz, H-3), 4.41 (d, J = 5.8 Hz, H-11), 5.34 (d, J = 6.0 Hz, H-8) and 5.78 (d, J = 3.1 Hz, H-4) (Table 1). The ^13^C-NMR and HSQC results revealed the presence of 24 carbon resonances attributable to six methyls, four methylenes, seven methines and seven quaternary carbons (including an oxygenated quaternary carbon at δ_C_ 65.3 (C-12), one olefinic carbon at δ_C_ 137.1 (C-9), three carbonyls at δ_C_ 172.3 (C-17), 172.1 (C-19) and 177.3 (C-1’) and two carbons at δ_C_ 49.8 (C-5) and 44.0 (C-6). A comparison of the NMR data of **1** with those reported suggested that **1** belonged to a group of trichothecenes sesquiterpenoids [12,13,14], except that the ester side chain at C-8 changed (Figure 1). The ^1^H-^1^H COSY correlations between H-2’ (δ_H_ 2.33) and H-5’ (δ_H_ 1.14) and between H-3’ (δ_H_ 1.65, 1.50) and H-4’ (δ_H_ 0.92) and HMBC correlations from H-4’ (δ_H_ 0.92) to C-2’ (δ_C_ 42.3), from H-3’ (δ_H_ 1.67, 1.50) to C-1’ (δ_C_ 177.3) and C-2’ (δ_C_ 42.3), and from H-5’ (δ_H_ 1.14) to C-1’ (δ_C_ 177.3) revealed that **1** contained the 2-methylbutyryl group (Figure 2). In addition, HMBC correlations (Figure 2) from H-8 (δ_H_ 5.34) to C-1’ (δ_C_ 177.3) showed that the ester group was also linked to the C-8 position. 

**Figure 3:**
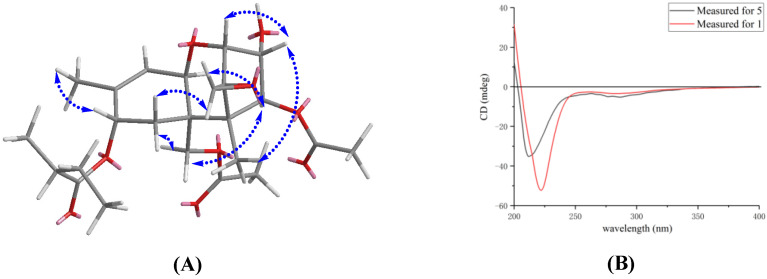
Key NOESY correlations (blue double arrow) of compound **1** (**A**); experimental ECD spectra of **1** (red line) and **5** (black line) in MeOH (**B**).

The relative configuration of **1** was established by analysis of NOESY correlations (Figure 3A). The observed NOESY correlations from H-11 (δ_H_ 4.41) to H-4 (δ_H_ 5.78), from H-15 (δ_H_ 4.34, 4.12) to H-4 (δ_H_ 5.78) and from H-7α (δ_H_ 1.85) to H-15 (δ_H_ 4.34, 4.12) indicated the H-11, H-4 and H-15 were in the α orientation. Furthermore, the NOESY cross-peaks of H-7β (δ_H_ 2.43) / H-13 (δ_H_ 3.02, 2.85), H-2 (δ_H_ 3.54) / H-3 (δ_H_ 4.22) / Me-14 (δ_H_ 0.72) positioned these protons on the other side. It is known that oxidations at C-8 in trichothecenes were stereospecific in Fusarium species [13]. The oxygen function at C-8 in the trichothecene ring normally resulted in an oxygen function with an 8α-configuration. Thus, the relative configuration of **1** was determined, which was consistent with known trichothecenes reported previously. 

Moreover, the absolute configuration of **5** was previously confirmed on the basis of single-crystal X-ray analysis [14]. Therefore, to determine the stereochemistry of **1**, we resorted to the ECD analysis by comparison with **5** (Figure 3B). The ECD spectrum of **1** (220 nm, Δε −1.86) displayed a similar negative cotton effect to that of **5** (211 nm, Δε −1.00). Therefore, the absolute stereochemistry of **1** is assumed to be the same as **5**. As far as we know, the trichothecenes that have been reported all had the same configuration [15,16]. It was given the name 8-(2-methylbutyryl)-neosolaniol (NT-3). The structures of the four known compounds were identified as 8-propionylneosolaniol (**2**) [17], 8-n-butyrylneosolaniol (**3**) [17], 8-isobutyrylneosolaniol (**4**) [17] and 8-n-pentanoylneosolaniol (**5**) [14] (Figure 1).

### 3.2. Anti-Tumor Activities of Compounds ***1***–***5***

The anti-tumor effects of isolated compounds **1**–**5** on Huh-7 and MRMT-1 were evaluated by CCK-8 assay. The results indicated all compounds showed strong inhibition of the growth of Huh-7 and MRMT-1 cells compared to the T-2 toxin. Surprisingly, **3** significantly decreased the cell viability of Huh-7 cells and MRMT-1 cells with the IC_50_ values of 265.9 nM and 279.4 nM, respectively (Table 2). These data indicated that **3** exhibited a more destructive effect in Huh-7 cells even than T-2 toxin. Due to the strong reduction in Huh-7 cells viability even at a very low concentration, the mechanism of action of **3** on Huh-7 cells was further studied. 

### 3.3. Effects of Compound ***3*** Inducing Cell Cycle Arrest in Huh-7 Cells 

**Figure 4:**
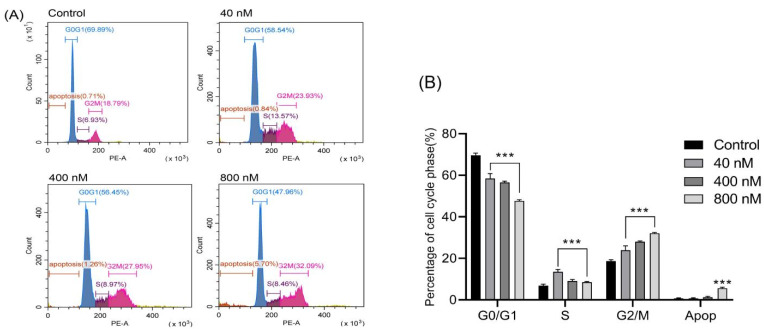
Effects of compound **3** on cell cycle distribution of Huh-7 cells determined by flow cytometry. (**A**) Histograms of cell cycle distribution after treatment with 0, 40, 400 and 800 nM compound **3** for 24 h. (**B**) Percentage of Huh-7 cell in G0/G1, S and G2/M phases after treatment with compound **3**. All data are the mean ± SD from three independent experiments. *** *p* < 0.001 vs. control group.

We investigated the changes in the cell cycle phase distribution on the basis of DNA content in Huh-7 cells by flow cytometry analysis using PI staining. A cell-cycle assay was performed by treating Huh-7 cells at various concentrations of **3** (0, 40, 400 and 800 nM) for 24 h, and the observed FACS histogram statistics are shown in Figure 4. In the untreated control group, the G2/M phase cells percentage was only 18.79%, which increased to 23.93%, 27.95% and 32.09% in groups treated with different concentrations of **3**. Therefore, the result suggested that **3** had a conspicuous effect on the cell cycle and compelled the cells to get arrested in the G2/M phase in a dose-dependent manner. 

### 3.4. Effects of Compound ***3*** Inducing Apoptosis of Huh-7 Cells

**Figure 5:**
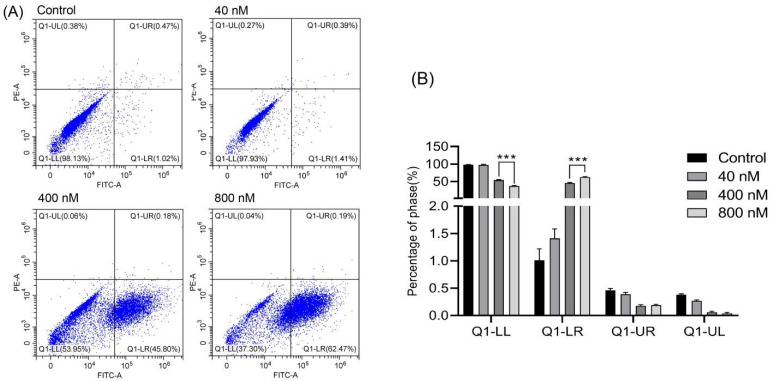
Effects of compound **3** to promote apoptosis of Huh-7 cells. Flow cytometry was used to detect the apoptotic cells by Annexin V/PI double-staining assay. (**A**) The pro-apoptotic effect of compound **3** (0, 40, 400 and 800 nM) in Huh-7 cells was analyzed by flow cytometry. (**B**) Apoptosis rates of the control group and compound group. Each value is the mean ± SD of three experiments. *** *p* < 0.001 vs. control group.

Next, we checked whether the anti-tumor activity of **3** was due to the induction of apoptosis in Huh-7 cells. It is well known that cells that were undergoing apoptosis would shift from the viable quadrant (Q1-LL) to the early apoptosis quadrant (Q1-LR) and eventually end up in the late apoptosis quadrant (Q1-UR). On the other hand, cells that underwent necrosis would shift from the viable quadrant (Q1-LR) to the late necrosis quadrant (Q1-UL). Cell apoptosis assay was performed with an Annexin V-FITC and PI double staining. After treatment with **3** for 36 h at different concentrations (0, 40, 400 and 800 nM), the distribution of cells undergoing apoptosis or necrosis was analyzed by flow cytometry assay. The results showed that the cells incubated with different concentrations of **3** for 36 h underwent apoptosis (Figure 5). As concentration increased, the rate of cell apoptosis significantly increased (62.47% for 800 nM treated with **3** and 1.02% for blank control), suggesting that **3** could significantly induce cell apoptosis in a dose-dependent manner. 

### 3.5. Effects of Compound ***3*** on Inducing Depolarization of Mitochondrial Membrane Potential and Generation of ROS

**Figure 6:**
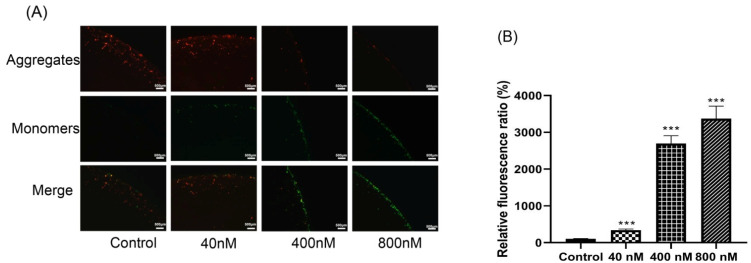
(**A**) Changes in mitochondrial membrane permeabilization in compound **3** treated Huh-7 cells by fluorescence microscopy. (**B**) The level of ROS after treatment with 0, 40, 400 and 800 nM of **3** for 24 h. The average levels of ROS were expressed as mean ± SD from three different experiments. *** *p* < 0.001 vs. control values.

To verify whether the mitochondria-mediated signaling pathway was involved in the induced apoptosis of **3**, MMP was measured by use of an MMP assay kit with JC-10 after treatment with **3**. In the normal mitochondria, the JC-10 can be aggregated and emit red fluorescence. With the depolarization of **3**, JC-10 would form monomers that could produce green fluorescence. A significant increase was observed in the intensity of the green fluorescence in Huh-7 cells, indicating that the MMP decreased after treatment with **3** (Figure 6A). The color change from red to green accompanied by increased doses of **3** indicated the increased mitochondrial injury. 

It has been shown in previous reports that ROS is involved in the mitochondria-mediated apoptosis pathway induced by multiple toxins [18]. Mitochondrial damage led to impaired oxidative phosphorylation and electron transport chain, which resulted in excessive ROS production and release from mitochondria. To determine whether **3** triggered ROS generation in Huh-7 cells, the ROS level was measured using 2’,7’-dichlorodihydrofluoresce in diacetate (DCFH-DA). When compared with the control group, the concentration assay of ROS levels showed a significant increase by treatment with **3** in a dose-dependent manner (Figure 6B).

### 3.6. Effect of Compound ***3*** on the Expression of Apoptosis-Related Proteins

**Figure 7:**
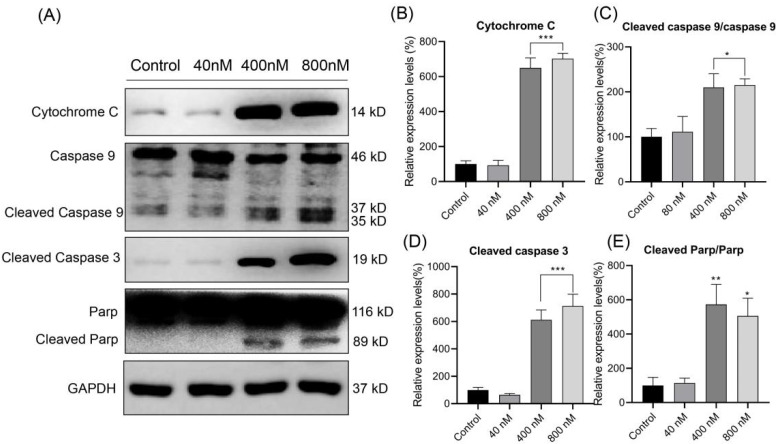
The effects of 3 on the expressions of cytochrome C, caspase-9, cleaved caspase-9, cleaved caspase-3, PARP and cleaved PARP evaluated via Western blot (**A**). Relative expressions of the proteins were normalized to GAPDH (**B**–**E**). * *p* < 0.05; ** *p* < 0.01; *** *p* < 0.001 vs. control values.

To further evaluate if **3** induced cell apoptosis by activating the mitochondrial pathway, Western blot analysis was used to detect the expression levels of mitochondrial apoptosis pathway-related proteins, as shown in Figure 7. To assess if the equilibrium of mitochondrial fusion was disturbed and caused cellular apoptosis, cytochrome C release was tested. As expected, the protein expression was higher in compound **3** groups. In addition, the expression level of cleaved caspase 9 was markedly increased as well. Moreover, as the concentration of **3** increased, the protein expression levels of cleaved caspase-3 and cleaved PARP were significantly increased. Altogether, our results indicated that **3** induced apoptosis via a caspase-dependent mechanism.

## 4. Discussion

During our research on anti-tumor secondary metabolites from endophytic fungi, one novel and four known trichothecene metabolites belonged to the type A trichothecene mycotoxin family [8]. T-2 toxin was the earliest investigated member of type A trichothecene, which was one of the most cytotoxicity trichothecenes up to now [18]. However, its anti-tumor activity has not been reported in Huh-7 cells. In this study, surprisingly, compound **3** exhibited lower IC_50_ values compared to T-2 toxin against Huh-7 cells, which was reported for the first time. Therefore, further mechanism experiments investigating the effects of compound **3** on Huh-7 cell are important. 

Cell cycle progression is tightly linked with cell proliferation [19]. The cell cycle is mainly adjusted by checkpoints which are activated by DNA damage to assure the exact progress of the cell cycle. In this condition, the growth arrest due to checkpoints allows cellular DNA damage repair; otherwise, the cell is eliminated by apoptosis [20,21]. When DNA is damaged, the G2/M checkpoint is a barrier that inhibits the cell’s progress entry into the M phase [22]. When cells possess an unrepaired DNA from S or G1 phases or partially replicated chromatin from the S phase. In this case, it is necessary to inhibit cell progress before mitotic entry [23]. Due to DNA damage, the cells activate an apoptotic program and halt mitotic during the metaphase [24]. In this study, our result demonstrated that the growth inhibitory effect of the **3** on Huh-7 cells was associated with a G2/M arrest and cell cycle progression.

Cell cycle could help to regulate cell growth, which played an important role in apoptosis. To investigate if the cell death caused by **3** was induced by apoptotic activity, the Annexin V-FITC/PI assay was used. This technique detects early apoptosis by means of monitoring the translocation of phosphatidylserine from the inner side of the cell membrane to the outer layer [25,26]. The quantification means of apoptotic cells are measured Annexin V-FITC binding to the externalized phosphatidylserine. Propidium iodide (PI) was added to distinguish between viable, early apoptotic, late apoptotic and necrotic cells [26,27]. After treating the Huh-7 cells with different concentrations of **3** for 36 h, the early apoptotic rate was significantly increased compared to the control group. The analysis of Huh-7 cells by flow cytometry revealed that treatment with the **3** induced a shift in the cell population toward apoptosis.

Apoptosis was often accompanied by a decrease in mitochondrial membrane permeabilization [28]. The decrease in mitochondrial membrane permeabilization was widely regarded as one of the earliest events of apoptosis [29]. It is crucial to maintain the mitochondrial membrane potential for mitochondrial function [30,31]. A mitochondrial membrane potential measurement with the membrane potential-sensitive dye JC-10 was conducted to confirm whether the mitochondria-mediated signaling pathway was involved in **3**-induced apoptosis. In our study, increased exposure to **3** led to a significant decrease in mitochondrial transmembrane potential, confirming that apoptosis was triggered through the mitochondrial pathway.

Mitochondria were the major cellular organelles that produced ROS. Furthermore, excessive ROS was involved in the damage of the mitochondrial membrane and the initiation of apoptotic tissue damage [32,33]. Thus, the effects of **3** on the level of ROS were investigated by the DCFH-DA method [34]. A significant dose-dependent increase in intracellular ROS in Huh-7 cells treated by **3** gave evidence that **3** could inhibit mitochondrial dysfunction.

Subsequently, we explored the molecular mechanisms underlying the regulation of cell apoptosis processes. Apoptosis was a form of normal programmed cell death and a complex process that could be triggered by many different factors [35,36]. Numerous studies have shown that the apoptotic pathway was categorized into the extrinsic apoptotic and intrinsic apoptotic pathways [37]. The mitochondria-initiated intrinsic pathway required the release of cytochrome C and promoted the caspase-activating apoptosome, a complex that induced the activation of caspase-9 and initiated the apoptotic caspase cascades [35]. Caspases are both the initiators and executors of cell death, which are the center of the apoptosis mechanism [38]. Moreover, activation of caspase was regarded as a typical indication of apoptosis [39]. Caspase-3 is regarded as the apoptosis promoter [40]. Thus, cytochrome C could activate caspase-9 and caspase-3 and stimulate the proteolytic cleavage of PARP, resulting in cell apoptosis. Taken together, our results indicate that **3** induced apoptosis via a caspase-dependent mechanism. Eventually, these studies offer vital information on the apoptosis and anti-tumor of trichothecenes, which could lead to further studies dedicated to this topic. Additionally, this research work may give an insight into the discovery of trichothecenes-derived new drug leads. In the future, we will further investigate if compound **3** could be developed as an anti-tumor agent through more in vivo experiments.

## 5. Conclusions

In summary, five secondary metabolites were isolated from *F. sporotrichioides*, including one new trichothecene. Among these compounds, **3** displayed significantly cytotoxic activity against Huh-7 cells by cell viability assay. The mechanism responsible for the cytotoxicity of **3** was further studied. Flow cytometry revealed that **3** could induce G2/M arrest and apoptosis. Further studies showed a significant decrease in mitochondrial membrane permeabilization and a significant increase in ROS generation, which led to the activation of caspase cascades (caspase-3 and caspase-9) and subsequent cleavage of PARP fragment. These results indicated that **3** induced cell apoptosis in Huh-7 cells via the mitochondria-mediated apoptotic signaling pathway.

## Figures and Tables

**Table 1:** ^1^H-NMR and ^13^C-NMR data for compound **1** (in CD_3_OD, 400 MHz for ^1^H-NMR and 100 MHz for ^13^C-NMR).

No.	*δ*_H_ *J* (Hz)	*δ*_C_	No.	*δ*_H_ *J* (Hz)	*δ*_C_
2	3.54, d, 4.9	80.4, CH	14	0.72, s	6.7, CH_3_
3	4.22, dd, 4.9, 3.1	77.8, CH	15	4.34, d, 12.5	65.9, CH_2_
4	5.78, d, 3.1	83.9, CH		4.12, d, 12.5	
5		49.8, C	16	1.74, s	20.2, CH_3_
6		44.0, C	17		172.3, C=O
7	2.43, dd, 15.3, 6.0	29.0, CH_2_	18	2.08, s	20.6, CH_3_
	1.85, d, 15.3		19		172.1, C=O
8	5.34, d, 6.0	69.1, CH	20	2.05, s	20.9, CH_3_
9		137.1, C	1’		177.3, C=O
10	5.80, d, 5.8	124.8, CH	2’	2.33, m	42.3, CH
11	4.41, d, 5.8	68.2, CH	3’	1.65, dq, 13.6, 7.4	27.6, CH_2_
12		65.3, C		1.50, dq, 13.6, 7.4	
13	3.02, d, 3.9	47.7, CH_2_	4’	0.92, t, 7.4	11.7, CH_3_
	2.85, d, 3.9		5’	1.14, d, 7.0	16.5, CH_3_

Proton coupling constants (*J*) in Hz are given in parentheses. The assignments were based on ^1^H-^1^H COSY, HSQC and HMBC experiments.

**Table 2:** Anti-tumor activities of compounds **1**–**5**.

Cell Lines	IC_50_ (nM)					
	1	2	3	4	5	T-2 Toxin
Huh-7	2202.7	890.7	265.9	479.7	676.4	357.1
MRMT-1	1739.0	679.8	279.4	462.4	577.4	122.0

## Data Availability

Data are included in the article/Supplementary Materials.

## Supplementary Materials

The following supporting information can be downloaded at: https://www.mdpi.com/article/10.3390/biom12060778/s1, Figure S1: ^1^H NMR (400 MHz, CD_3_OD) spectrum of 8-(2-methylbutyryl)-neosolaniol (**1**); Figure S2: ^13^C NMR (100 MHz, CD_3_OD) spectrum of 8-(2-methylbutyryl)-neosolaniol (**1**); Figure S3: ^1^H-^1^H COSY (600 MHz, CD_3_OD) spectrum of 8-(2-methylbutyryl)-neosolaniol (**1**); Figure S4: HSQC (600 MHz, CD_3_OD) spectrum of 8-(2-methylbutyryl)-neosolaniol (**1**); Figure S5: HMBC (600 MHz, CD_3_OD) spectrum of 8-(2-methylbutyryl)-neosolaniol (**1**); Figure S6: NOESY (600 MHz, CD_3_OD) spectrum of 8-(2-methylbutyryl)-neosolaniol (**1**); Figure S7: HRESIMS spectrum of 8-(2-methylbutyryl)-neosolaniol (**1**); Table S1: The ^1^H NMR and ^13^C NMR spectroscopic data of all known compounds.
