# Supplementary material for: Structure Elucidation and Anti-Tumor Activities of Trichothecenes from Endophytic Fungus Fusarium sporotrichioides
Source: Biomolecules. 2022 Jun 2;12(6):778. doi: 10.3390/biom12060778 (PMC9220965; doi:10.3390/biom12060778)
Supplement: Supplementary file 1 [file biomolecules-12-00778-s001.zip › biomolecules-1757489-supplementary.pdf]

Supplementary data:

Figure S1. ^{1}H NMR (400 MHz, CD_3OD) spectrum of 8-(2-methylbutyryl)-neosolaniol (**1**).

Figure S2. ^{13}C NMR (100 MHz, CD_3OD) spectrum of 8-(2-methylbutyryl)-neosolaniol (**1**).

Figure S3. ^{1}H-^{1}H COSY (600MHz, CD_3OD) spectrum of 8-(2-methylbutyryl)-neosolaniol (**1**).

Figure S4. HSQC (600MHz, CD_3OD) spectrum of 8-(2-methylbutyryl)-neosolaniol (**1**).

Figure S5. HMBC (600MHz, CD_3OD) spectrum of 8-(2-methylbutyryl)-neosolaniol (**1**).

Figure S6. NOESY (600MHz, CD_3OD) spectrum of 8-(2-methylbutyryl)-neosolaniol (**1**).

Figure S7. HRESIMS spectrum of 8-(2-methylbutyryl)-neosolaniol (**1**).

Table S1. The ^{1}H NMR and ^{13}C NMR spectroscopic data of all known compounds (**2-5**).

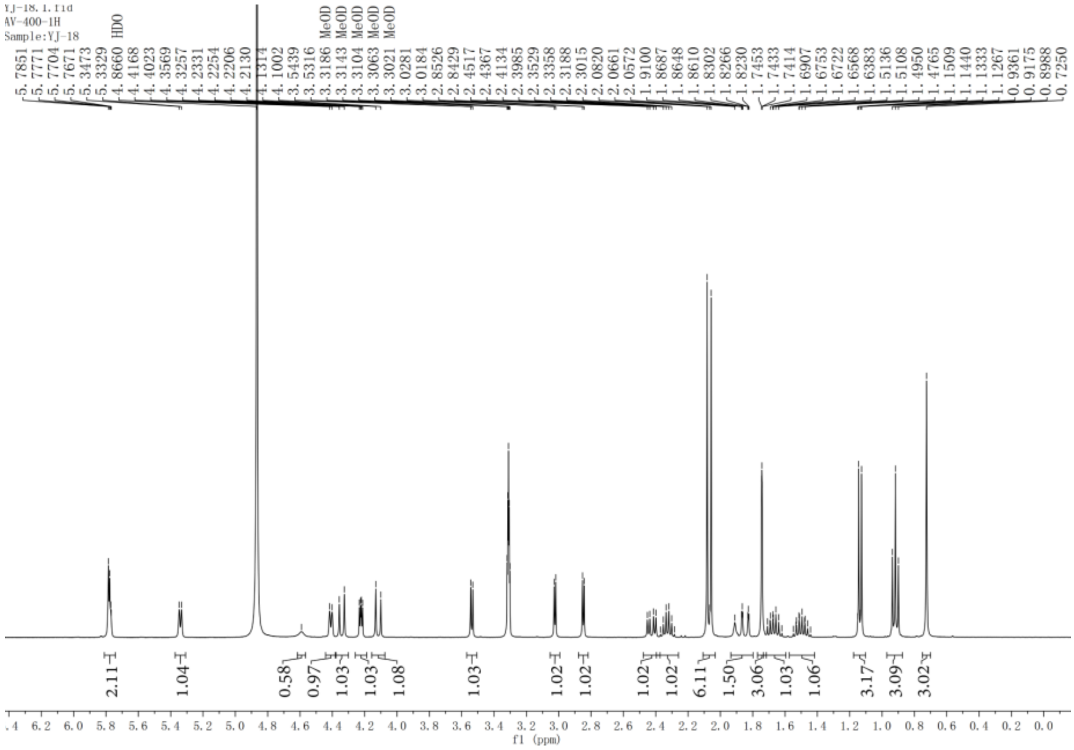

Figure S1. ^{1}H NMR (400 MHz, CD_3OD) spectrum of 8-(2-methylbutyryl)-neosolaniol (**1**).

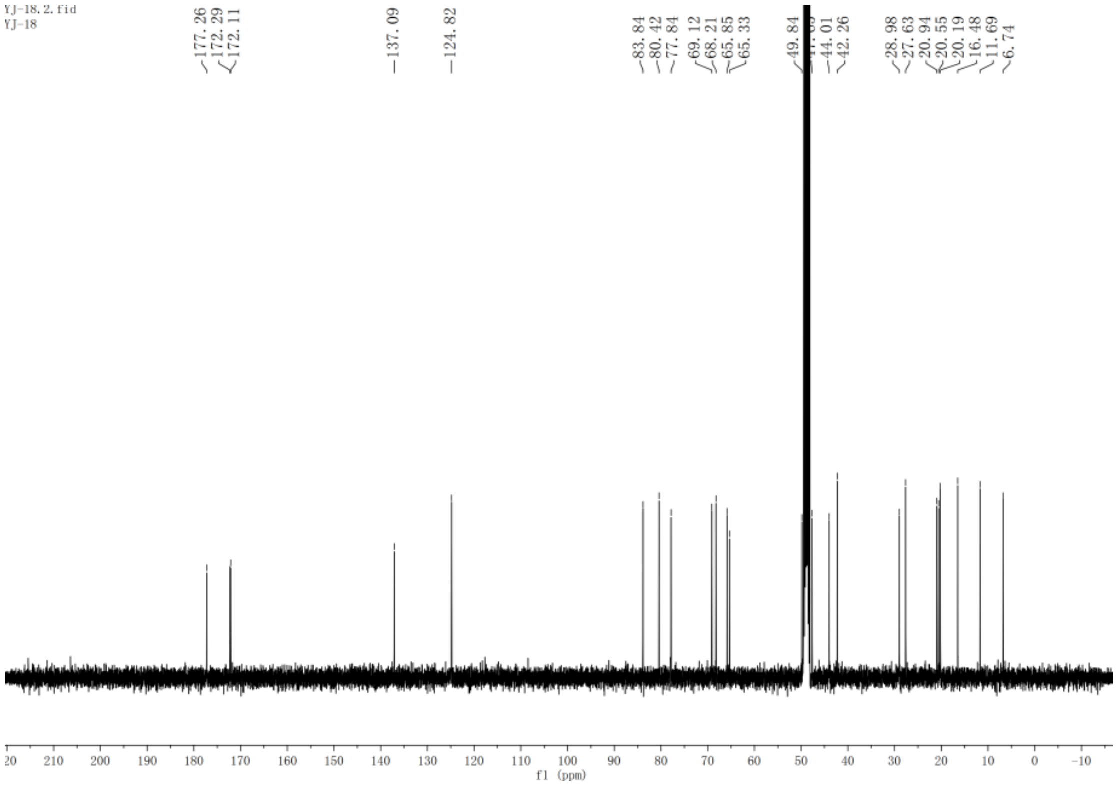

Figure S2. ^{13}C NMR (100 MHz, CD_3OD) spectrum of 8-(2-methylbutyryl)-neosolaniol (**1**).

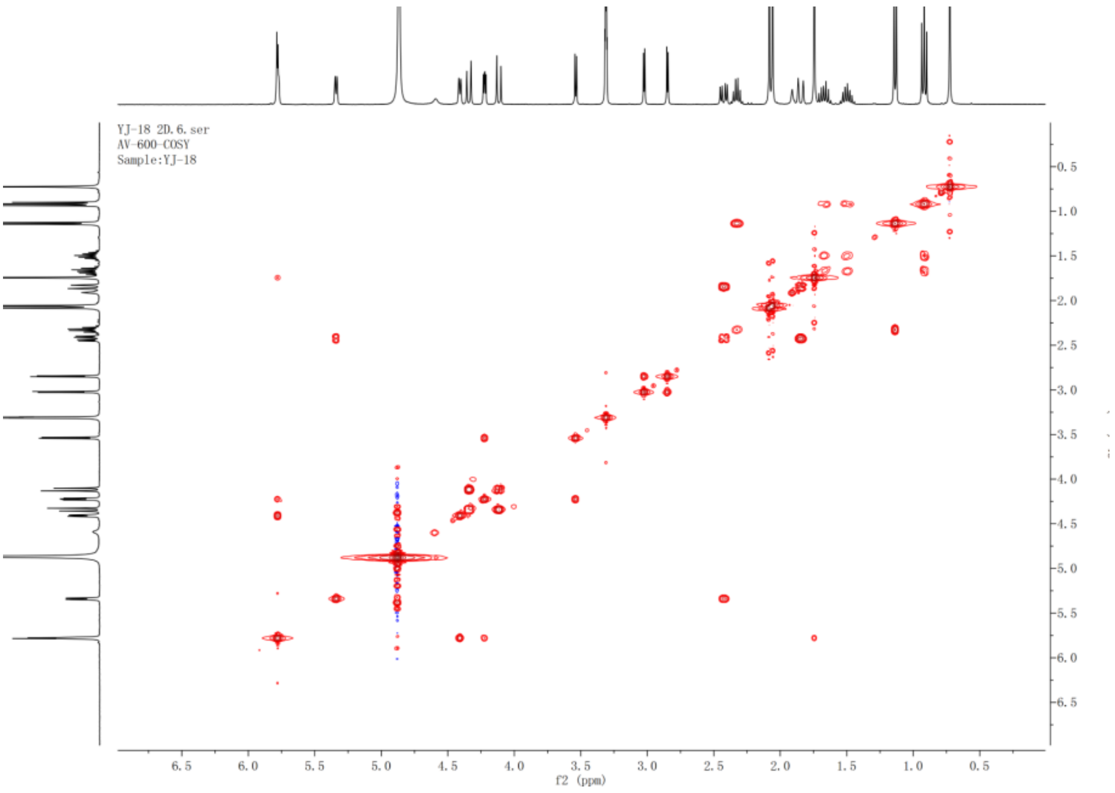

Figure S3. ^{1}H-^{1}H COSY (600MHz, CD_3OD) spectrum of 8-(2-methylbutyryl)-neosolaniol (**1**).

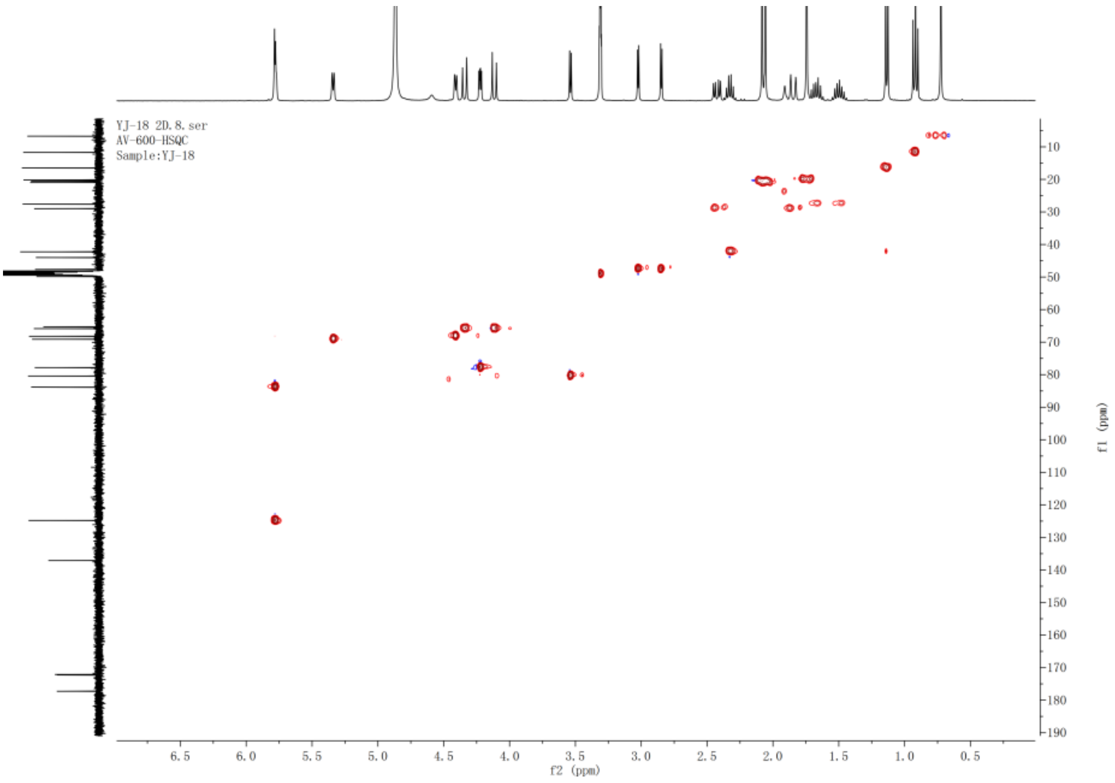

Figure S4. HSQC (600MHz, CD_3OD) spectrum of 8-(2-methylbutyryl)-neosolaniol (**1**).

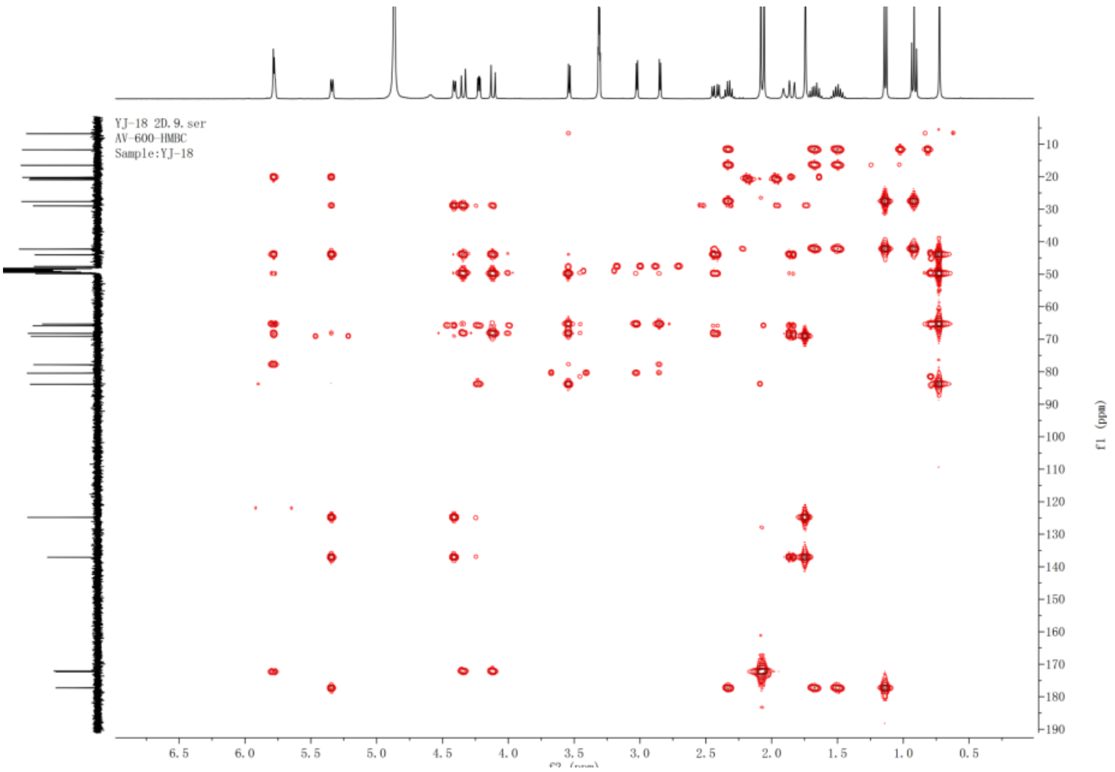

Figure S5. HMBC (600MHz, CD_3OD) spectrum of 8-(2-methylbutyryl)-neosolaniol (**1**).

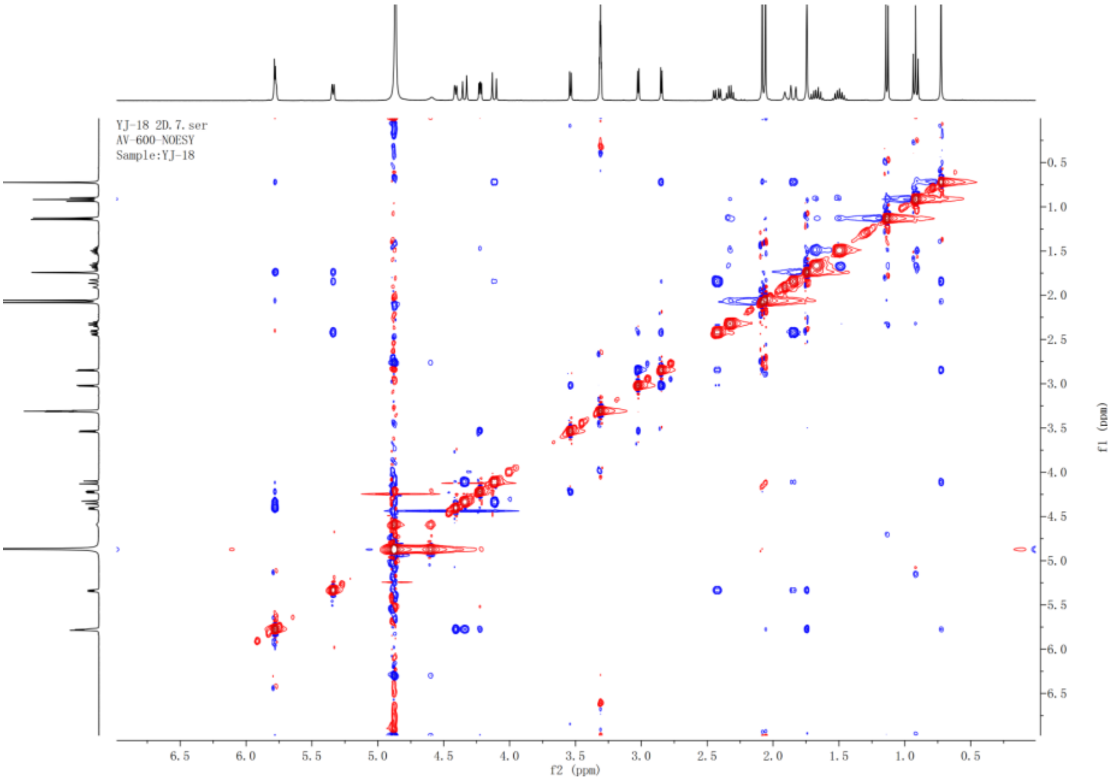

Figure S6. NOESY (600MHz, CD_3OD) spectrum of 8-(2-methylbutyryl)-neosolaniol (**1**).

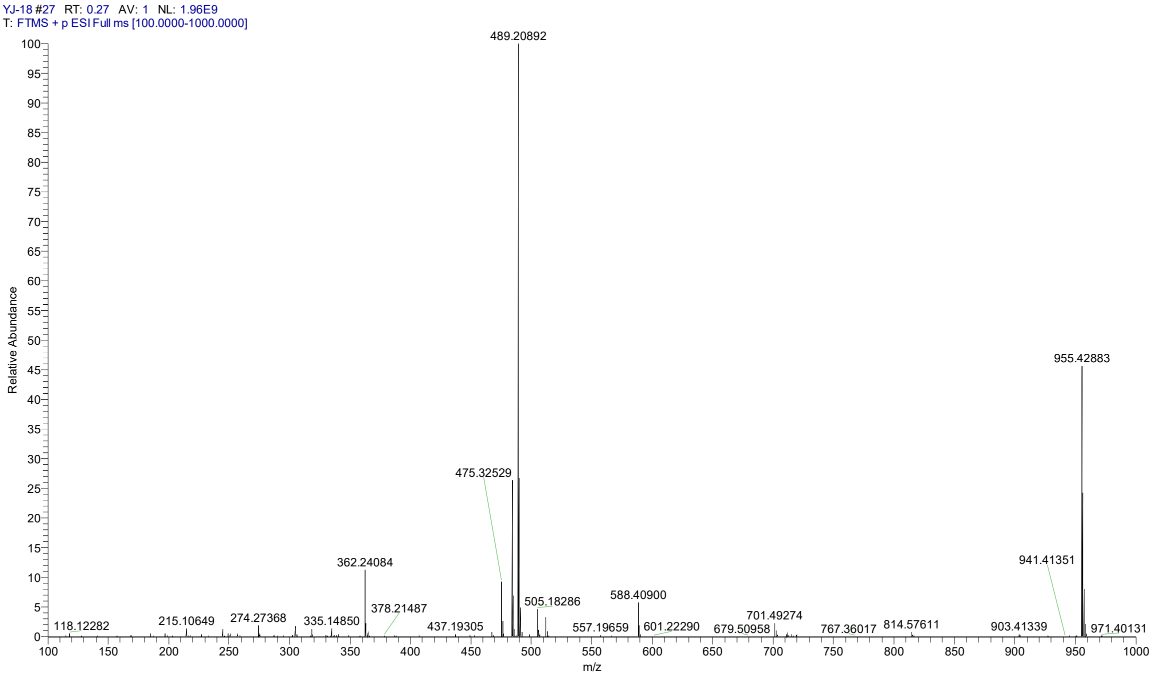

Elemental composition search on mass 489.20892

m/z= 484.20892-494.20892

m/z	Theo. Mass	Delta (ppm)	RDB equiv.	Composition
489.20892	489.20950	-1.18	13.0	$C_{23}H_{28}O_4N_7Na$
	489.20950	-1.19	7.5	$C_{24}H_{34}O_9Na$
	489.20816	1.55	8.0	$C_{22}H_{32}O_8N_3Na$
	489.20816	1.56	13.5	$C_{21}H_{26}O_3N_{10}Na$
	489.21084	-3.93	12.5	$C_{25}H_{30}O_5N_4Na$
	489.20682	4.30	8.5	$C_{20}H_{30}O_7N_6Na$
	489.21135	-4.96	0.0	$C_{11}H_{32}O_{11}N_9Na$

Figure S7. HRESIMS spectrum of 8-(2-methylbutyryl)-neosolaniol (**1**).

Table S1. The ^{1}H NMR and ^{13}C NMR spectroscopic data of all known compounds.

No.	Compound 2 [a]		Compound 3 [a]		Compound 4 [a]		Compound 5 [a]	
	δ_H J (Hz)	δ_C	δ_H J (Hz)	δ_C	δ_H J (Hz)	δ_C	δ_H J (Hz)	δ_C
2	3.53 (1H, d, 4.9)	80.4	3.53 (1H, d, 5.0)	80.4	3.54 (1H, d, 5.0)	80.4	3.53 (1H, d, 5.0)	80.4
3	4.25 (1H, dd, 4.9, 3.1)	77.8	4.23 (1H, dd, 5.0, 3.1)	77.8	4.22 (1H, dd, 5.0, 3.1)	77.9	4.23 (1H, dd, 5.0, 3.2)	77.8
4	5.66 (1H, d, 3.1)	83.9	5.68 (1H, d, 3.1)	83.9	5.77 (1H, d, 3.1)	83.9	5.68 (1H, d, 3.2)	83.9
5		50.0		50.0		49.9		50.0
6		44.1		44.1		44.0		44.1
7	2.32 (1H, dd, 15.3, 6.0)<br>1.97 (1H, d, 15.3)	28.4	2.38 (1H, dd, 15.2, 5.9)<br>1.94 (1H, d, 15.2)	28.5	2.41 (1H, dd, 15.3, 5.7)<br>1.89 (1H, d, 15.3)	28.8	2.38 (1H, dd, 15.3, 5.9)<br>1.95 (1H, d, 15.3)	28.5
8	5.31 (1H, d, 6.0)	69.4	5.33 (1H, d, 5.9)	69.2	5.32 (1H, d, 5.7)	69.2	5.32 (1H, d, 5.9)	69.3
9		137.2		137.1		137.2		137.1
10	5.76 (1H, d, 6.0)	124.7	5.76 (1H, d, 6.0)	124.8	5.79 (1H, d, 6.0)	124.7	5.76 (1H, d, 6.2)	124.8
11	4.31 (1H, d, 6.0)	68.3	4.35 (1H, d, 6.0)	68.3	4.39 (1H, d, 6.0)	68.2	4.35 (1H, d, 6.2)	68.3
12		65.3		65.3		65.3		65.3
13	3.02 (1H, d, 3.9)<br>2.84 (1H, d, 3.9)	47.6	3.02 (1H, d, 3.9)<br>2.85 (1H, d, 3.9)	47.6	3.02 (1H, d, 3.9)<br>2.84 (1H, d, 3.9)	47.6	3.02 (1H, d, 3.9)<br>2.85 (1H, d, 3.9)	47.6
14	0.74 (3H, s)	6.9	0.73 (3H, s)	6.9	0.73 (3H, s)	6.8	0.73 (3H, s)	6.9
15	4.35 (1H, d, 12.4)<br>4.10 (1H, d, 12.4)	65.6	4.34 (1H, d, 12.4)<br>4.11 (1H, d, 12.4)	65.7	4.35 (1H, d, 12.5)<br>4.10 (1H, d, 12.5)	65.8	4.32 (1H, d, 12.5)<br>4.11 (1H, d, 12.5)	65.7
16	1.74 (3H, s)	20.9	1.74 (3H, s)	19.3	1.74 (3H, s)	20.1	1.74 (3H, s)	20.1
17		172.4		172.4		172.3		172.4
18		172.0		172.0		172.1		172.0
1'		175.3		174.4		177.7		174.6
2'	2.38 (2H, m)	28.3	2.26 (2H, t, 7.3)	37.0	2.50 (2H, m)	35.2	2.28 (2H, td, 7.4, 2.5)	34.9
3'	1.12 (3H, t, 7.6)	9.2	1.64 (2H,m)	20.9	1.16 (3H,d, 3.9)	19.3	1.59 (2H,m)	28.0
4'			0.95 (3H, t, 7.4)	13.8	1.15 (3H,d, 3.9)	18.8	1.36 (2H,m)	23.1
5'							0.93 (3H, d, 7.4)	13.8
17-CH_3	2.03 (3H, s)	20.5	2.04 (3H, s)		2.05 (3H, s)		2.04 (3H, s)	
18-CH_3	2.08 (3H, s)	20.1	2.08 (3H, s)		2.08 (3H, s)		2.08 (3H, s)	

[a] Measured in CD_3OD at 400 MHz for ^{1}H NMR and 100 MHz for ^{13}C NMR.
